# The Current Situation of Esophageal Cancer Staging and Perioperative Strategies Determination in Central and Southern China: A Cross Sectional Survey

**DOI:** 10.3389/fonc.2019.01098

**Published:** 2019-10-22

**Authors:** Di Lu, Xiguang Liu, Siyang Feng, Xiaoying Dong, Xiaoshun Shi, Pengfei Ren, Dingwei Diao, Hua Wu, Gang Xiong, Haofei Wang, Mei Li, Shuan Rao, Daniela Molena, Abraham J. Wu, Kaican Cai

**Affiliations:** ^1^Department of Thoracic Surgery, Nanfang Hospital, Southern Medical University, Guangzhou, China; ^2^Department of Surgery, Memorial Sloan Kettering Cancer Center, New York, NY, United States; ^3^Department of Radiation Oncology, Memorial Sloan Kettering Cancer Center, New York, NY, United States

**Keywords:** esophageal cancer, TNM, perioperative strategies, central and southern China, current situation

## Abstract

**Purpose:** We aim to investigate the current esophageal cancer staging according to the 7th edition TNM classification for esophageal carcinoma proposed by American Joint Committee on Cancer (AJCC) among oncology-related physicians in China.

**Methods:** A specifically-designed 14-item questionnaire was distributed to 366 doctors who were working with esophageal cancer patients. We collected and analyzed the feedbacks and explored the possible associations within different departments, including thoracic surgery, the internal medicine of gastroenterology, oncology, and/ radiotherapy in eight different hospitals from central and southern China.

**Results:** Among all the responses, 31.42% of them were from thoracic surgery department, 40.44% were from oncology and/or radiation therapy and 28.14% were from the internal medicine of gastroenterology, respectively. Surprisingly, in total 66.12% of all the physicians were unaware that the 7th edition of esophageal carcinoma TNM classification was released in 2009; only 21.86 and 16.67% of physicians recognized cervical nodes and celiac nodes as regional lymph nodes. Furthermore, 67.21% physicians didn't know that tumor location, histologic grade, and histopathology were accepted as new prognostic factors in the latest TNM system; and 51.37% physicians could not determine the correct TNM classification of esophagogastric junction cancers. Intriguingly, over 50% of them could still design appropriate perioperative strategies.

**Conclusions:** The 7th edition of the TNM classification for esophageal carcinoma is poorly recognized and understood in central and southern China, which might contribute to the relatively low rates of appropriate perioperative procedures applied for esophageal cancer patients.

## Introduction

Esophageal cancer is the eighth most common cancer worldwide with an estimated incidence of 6.5 per 100,000 in 2012 (3.2% of all cancer occurrence), and the sixth most common cause of cancer death with a roughly mortality of 5.7 per 100,000 (4.9% of all cancer-related death). Mortality variation shows apparent geographical difference with the highest one occurring in Eastern Asia (14.1 per 100,000) (1). Particularly, esophageal cancer is the 5th most common cancer (22.16 per 100,000) and 4th most common cause (16.64 per 100,000) of cancer related death in China (2); indicating China has a more severe esophageal cancer burden compared to other regions.

The prognosis for early stage esophageal cancer patients is significantly superior to that of intermediate and late stage patients. However, the overall survival of esophageal cancer in China is very low due to the undeveloped early detection of esophageal cancer via endoscopy thus the majority of patients are diagnosed as the intermediate or late stages (3). Importantly, the poor prognosis might also be caused by clinicians' limited knowledge of esophageal cancer, for example, the TNM staging system, which is extremely important for corresponding treatments planning.

We realized that many physicians and surgeons from esophageal related departments, including thoracic surgery, the internal medicine of gastroenterology, oncology, and radiotherapy departments, with different levels of experience, were not fully aware of neither the 7th edition of TNM classification of esophageal carcinoma which was proposed by American Joint Committee on Cancer (AJCC) in 2009 (4), nor the 2nd edition of Chinese Guidance for Standardized Therapy for Esophageal Carcinoma (5). To our knowledge, there is no cross-sectional survey on the awareness of the esophageal cancer TNM staging system or perioperative strategies determination among Chinese clinicians so far. Therefore, we performed the current survey to explore the possible correlations between recognition of TNM system and perioperative procedures planning, with the ultimate goal to promote standard diagnosis and treatment for esophageal cancer in China.

## Methods

### Questionnaire

To obtain the first-hand data regarding current situation of esophageal cancer Staging and perioperative strategies in central and southern China, we carried out this cross-sectional study by generating a specific questionnaire. The questionnaire was developed by Di Lu, Siyang Feng, and Kaican Cai with help of twelve esophageal cancer experts and optimized based on two semi-structured pilot surveys. It was composed with 14 items and modified over ten times, which made it more acceptable to the responders. Finally, the 13th edition (Supplementary Data Questionnaire 1) with three sections and fourteen questions was applied in the current study and described as follows. Section 1: TNM staging (awareness of the 7th edition TNM staging system; classification of cervical nodes; classification of celiac nodes; awareness of new factors of the staging system; distinguishing between esophageal cancer and gastric cancer); Section 2: perioperative therapy (POT), (2 cycles of neo-adjuvant chemotherapy, 4 cycles of adjuvant chemotherapy, chemotherapy protocol (paclitaxel or platinum-based) for squamous cell cancer, dose of neo-adjuvant radiation); and Section 3: general opinion and access to staging system updates.

### Selection of Hospitals and Examinees

The survey was initiated in Guangdong province and most clinicians were from central and southern China. Particularly, the Chaoshan area, one of the representative areas with the highest incidence of esophageal cancer in China was enrolled in the current survey. The survey was carried out from May 2016 to December 2016, in total eight medical centers from Guangdong, Hunan, Henan, and Shanxi Provinces were selected, including Sun Yat-sen University Cancer Center, Nanfang Hospital of Southern Medical University, General Hospital of Guangzhou Military Region, Gaozhou People's Hospital, Shantou Central Hospital, Xiangya Hospital of Central South University, The First Affiliated Hospital of Zhengzhou University, and the Cancer Hospital of Shanxi Province. The examinees were from department of thoracic surgery, the internal medicine of gastroenterology, oncology, and radiation therapy, ranging from intern to professor. Informed consent was obtained from every responder before the survey.

### Data Collection

After he or she had agreed that the survey was anonymous and data were independently collected and would be published, each examinee was asked to complete the survey immediately upon receipt of the questionnaire without access to the internet, textbooks, and their colleagues, under the supervision of inspectors, to make sure the survey was firsthand and unbiased. The survey was carried out in an anonymous and independent fashion. Questionnaires were considered as valid if all questions were addressed properly according to the request.

### Statistical Analysis

The results were presented as counts or percentages. Statistical analysis was performed using SPSS version 22.0 (SPSS Inc., Chicago, IL, USA). Frequency tables were generated for relevant variables. Differences among several groups were analyzed by the chi-squared test. A two-sided *p*-value < 0.05 was considered as significant in all analyses.

## Results

In total of 401 questionnaires were distributed and 366 validated questionnaires were analyzed; the response rate was 91.3%. 31.42% of them were from the department of thoracic surgery, 40.44% from oncology (including radiation therapy) and 28.14% from the internal medicine of gastroenterology. Approximately a quarter of the examinees (25.14%) were senior attendings and professors, 25.41% were junior attendings, 21.58% were residents, and the rest were interns (27.87%). The regional details for all the examinees were summarized and presented in Table 1.

**Table 1:** The current situation of esophageal cancer staging in central and southern China (^*^P < 0.05).

**Variables**	**Total (%)**	**Aware-ness of the 7th edition (%)**	***P* value**	**Correct answer of cervical nodes (%)**	***P* value**	**Correct answer of celiac nodes (%)**	***P* value**	**Aware-ness of adding factors to the staging system (%)**	***P* value**	**Correct distinguishment of esophageal cancer from gastric cancer (%)**	***P* value**
Overall	100	38.25		21.86		16.67		32.79		48.63	
Area			0.33		<0.01^*^		<0.01^*^		<0.01^*^		<0.01^*^
Guang-dong	68.58	41.04		28.69		19.52		31.08		44.22	
Shanxi	13.66	36.00		2.00		4.00		24.00		64.00	
Hunan	2.19	37.50		25.00		62.50		75.00		87.50	
Henan	15.57	28.07		8.77		8.77		42.11		49.12	
Academic Level			0.015^*^		0.316		0.422		<0.01^*^		0.011^*^
Intern	27.87	26.47		23.53		15.69		38.24		36.27	
Resident	21.58	41.77		25.32		16.46		39.24		46.84	
Junior attending	25.41	48.39		13.98		16.13		31.18		53.76	
Senior attending and professor	25.14	38.04		25.00		18.48		22.83		58.70	
Department			<0.01^*^		<0.01^*^		<0.01^*^		<0.01^*^		0.011^*^
Thoracic surgery	31.42	59.13		17.39		25.22		43.48		58.26	
Oncology	40.44	22.97		29.05		14.86		25.00		48.65	
Gastro-enterology	28.14	36.89		16.50		9.71		32.04		37.86	

Regarding TNM staging, only 38.25% of the examinees knew this widely accepted staging system. Moreover, 33.88% of the examinees did not realize the 7th edition was the latest version by the time when this survey was conducted and 53.01% of them did not know this version was released in 2009. There were no differences between different areas regarding the low awareness of the TNM system, however, our data suggested that examinees from different academic levels (*p* = 0.015) or departments (*p* < 0.01) had significant difference in awareness of the latest TNM staging system. Of note, junior attendings knew the system best compared to other levels and surgeons from thoracic department were more familiar with the system than other physicians.

Overall, only 21.86 and 16.67% of all clinicians considered cervical nodes and celiac nodes as regional lymph nodes and there was a clear variation among examinees from different areas and departments (*p* < 0.01). Moreover, 67.21% were not aware that the new prognostic factors including tumor location, histological grade, and histopathology were added to the latest version of TNM system, not surprisingly, there was also significant difference among people from different areas, academic levels and departments regarding the new factors (*p* < 0.01).

Only 48.63% physicians could distinguish esophageal cancer staging from gastric cancer staging when they classified esophagogastric junction cancers according to the 7th edition of the TNM staging discipline. When we further analyzed the data in details, we found that difference of region, academic level, and department could induce significant difference in distinguishing esophageal cancer staging from gastric cancer staging.

**Figure 1:**
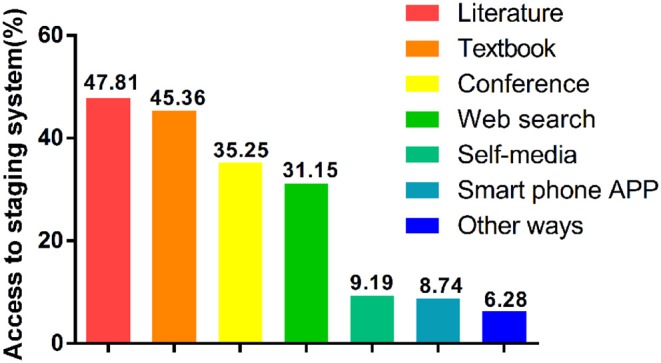
Proportion of access to the 7th edition of the esophageal cancer TNM staging system updates.

In addition, we also analyzed the method to access the 7th edition updates of these examinees, and the three major resources were academic literature (47.81%), textbooks (45.36%), and conferences (35.25%) (Figure 1). Surprisingly, we observed only <9% examinees got the updates from smart phone application, suggesting a lot of effort should be taken to improve this most efficient method to remind physicians with the latest updates.

As for perioperative therapy choice (Table 2), 53.28% of the examinees preferred two cycles of neo-adjuvant chemotherapy, 64.21% preferred four cycles of postoperative adjuvant chemotherapy, and 65.57% preferred a dose of 40 Gy for neo-adjuvant radiotherapy with suitable circumstances, these results were consistent with Chinese guideline and high-level randomized clinical trials results in this area (6). For pre- or postoperative adjuvant chemotherapy, 62.3% of examinees would choose paclitaxel and platinum rather than 5-fluorouracil and platinum for esophageal squamous cancer patients, furthermore, 70.49% of clinicians selected neo-adjuvant radio-chemotherapy but not chemotherapy alone to treat patients with local lymph node metastasis. When we looked into these results in more details, we frequently observed significant difference in selecting therapy procedures among physicians from different regions, academic levels and departments, indicating a standard treatment reference is lacking for esophageal cancer patients, which might partially explain the poor prognosis for this prevalent cancer in China.

**Table 2:** Summary of peri-operative therapy decision for esophageal cancer patients in central and southern China (^*^P < 0.05).

**Variables**	**Total (%)**	**2 cycles of neo-adjuvant chemo-therapy (%)**	***P* value**	**4 cycles of adjuvant chemo-therapy (%)**	***P* value**	**Chemo-therapy protocol (paclitaxel and platinum-based) for squamous cell cancer (%)**	***P* value**	**Dose of neo-adjuvant radiation (%)**	***P* value**
Overall	100	53.28		64.21		62.30		65.57	
Area			0.020^*^		0.227		<0.01^*^		<0.01^*^
Guangdong	68.58	49.00		65.74		61.35		63.35	
Shanxi	13.66	64.00		66.00		60.00		72.00	
Hunan	2.19	75.00		25.00		25.00		25.00	
Henan	15.57	59.65		61.40		73.68		75.44	
Academic level			<0.01^*^		0.663		0.314		0.220
Intern	27.87	35.29		60.78		65.69		66.67	
Resident	21.58	51.90		67.09		53.16		60.76	
Junior attending	25.41	62.37		67.74		58.06		72.04	
Senior attending and professor	25.14	65.22		61.96		70.65		61.96	
Department			<0.01^*^		<0.01^*^		0.068		<0.01^*^
Thoracic surgery	31.42	71.30		74.78		51.30		49.57	
Oncology	40.44	32.43		48.65		69.59		66.89	
Gastro-enterology	28.14	63.11		74.76		64.08		81.55	

Taken together, our survey revealed that the awareness of the 7th edition TNM staging system and POT decision making was significant different among different regions in central and southern China. Interestingly, physicians from different departments also showed marked difference in recognition of TNM system and POT determination, while thoracic surgeons usually performed better than clinicians from other departments according to our cross-sectional study.

## Discussion

Unlike many other cancers such as lung or colon cancer which drives massive research interest and dramatic improvement of treatment in last few decades, esophageal cancer is still one of the leading causes of cancer-related death with poor prognosis in China. One reason for this phenomenon is that most esophageal cancer patients in China are only diagnosed as locally advanced disease or unsuitable for radical resection at the first-time visit due to the lack of early detection via endoscopy (7, 8), therefore it is very challenging and difficult in most cases for esophageal cancer treatment. Endoscopic technologies have always been one of the most popular research interest for early detection (9). For instance, Lugol's iodine chromoendoscopy (LCE) was proved to be a useful tool to diagnose squamous cell neoplasia in high-risk individuals with a sensitivity of 46% and a specificity of 90% in 190 high-risk subjects (10). Importantly, a recent randomized trial showed that the overall accuracy of narrow band imaging (NBI) and LCE in detecting high grade dysplasia (HGD) or invasive squamous cell carcinoma was comparable (91.2 and 90.5%, respectively), but NBI was significantly more time-saving (11). In addition, the American Society for Gastrointestinal Endoscopy meta-analysis found that the pooled specificity and sensitivity of confocal laser endomicroscopy for diagnosing HGD were satisfying (77.3 and 90.3% respectively) (12). Besides these newly developed technologies, trans-nasal endoscopy (TNE) was identified to be more popular than conventional endoscopy in 63% of cases (13) without sacrificing sensitivity as compared to LCE (14, 15). Alternatively, apart from classical endoscopy methods, esophageal cell collection devices such as Cytosponge™ in diagnosing Barrett's Esophagus (BE) could achieve satisfying sensitivity and specificity (94 and 79.9%, respectively) (16). Moreover, several studies had shown that miRNAs in peripheral blood could efficiently distinguish BE patients from healthy individuals (17), whereas circulating tumor DNA (ctDNA) could be detected in the scenario of advanced esophageal cancer (18). Interestingly, a panel of breath volatile organic compounds was also applied to determine esophagogastric cancer (19). However, most of these newly developed technologies were not widely practiced for early esophageal cancer detection in Chinese population, thus we advocate a significant effort was required to promote these technologies in China.

Secondly, most research and therapy progress in esophageal cancer therapy is carried out in Western countries, yet there is a huge difference in esophageal cancer subtypes distribution between Western and Eastern countries. The majority of esophageal cancer patients in Western countries are adenocarcinoma, while in China over 90% esophageal cancer patients are squamous (20). Recent studies suggested that these two subtypes of esophageal cancer might be completely different in terms of histopathology, risk factors, and prognostic factors (21), therefore similar treatment might bring different therapeutic effects. Furthermore, it is still uncertain which genetic mutations are major driver of esophageal cancer, thus the establishment of *in vivo* system to recapitulate esophageal cancer development is still lacking, making the preclinical evaluation of targeted therapy or immunotherapy for esophageal cancer unfeasible.

Nevertheless, we believe other reasons, particularly in clinical, might contribute to the poor prognosis of esophageal cancer in China. As we frequently noticed divergence in diagnosis and therapy selection for esophageal cancer patients, we carried out the first cross sectional survey on the recognition of the 7th edition of the esophageal cancer TNM staging system and the current situation of perioperative therapy decision making in central and southern China. To our surprise, less than half of the examinees were aware of the 7th TNM staging system although more than half physicians could make the correct perioperative decision. The 7th edition of the TNM staging system for esophageal cancer is established based on large population study and identifies cervical and celiac lymph nodes as regional nodes. However, given the fact that the esophagus involves multiple regions, it might be confused to classify lymphatic metastasis when the primary tumor and metastatic lymph nodes are located in different regions. Indeed, according to our results, nearly half of the examinees thought that the 7th edition of the TNM staging system for esophageal cancer was debatable, as this latest staging system is more relying on patient data without highlighting the anatomic or biologic properties of esophageal cancer, therefore it is not completely convincing to all clinicians. The awareness of standard TNM staging system can influence correct therapy decision, indeed, our data suggested different levels physicians from different regions or departments showed inconsistence in recognition of the staging system, therefore the ratio of correct POT decision was also quite variable. As the 8th edition of the TNM staging system was released in October 2016 and has been practically used in clinical since January 2018, we advocate the promotion of this latest TNM system as soon as possible to improve the diagnosis and treatment of esophageal cancer in China.

We also admitted several limitations in the present survey. First, the selection of medical centers was not randomized and did not cover the entire country, though enrolled examinees were from those areas with high incidence of esophageal squamous cell cancer. Second, the sample size of the respondents might not be large enough to represent the accurate awareness and therapy decision making in China. Finally, the questionnaire included updates to the 7th edition and perioperative therapy for esophageal cancer but did not with full details of end-stage esophageal cancer therapy, which therefore does not represent the entire spectrum of treatment for esophageal cancer.

## Conclusions

In central and south China, the 7th edition of the AJCC TNM staging system has not been well-accepted and applied, and the current state of decision making for esophageal cancer is not satisfying. The promotion of standardized diagnosis and treatment for esophageal cancer is urgently required.

## Data Availability Statement

The raw data supporting the conclusions of this manuscript will be made available by the authors, without undue reservation, to any qualified researcher.

## Ethics Statement

This study was carried out in accordance with the recommendations of the ethical committee of Nanfang Hospital, Southern Medical University, with written informed consent from all subjects. All subjects gave written informed consent in accordance with the Declaration of Helsinki. The protocol was approved by the name of committee.

## Author Contributions

DL and KC designed the study. DL and XL were primarily responsible for analyzing the data and writing the manuscript. DL, SF, and KC designed the questionnaire. XD, XS, PR, and DD were responsible for data collection. HWu, GX, HWa, and ML recruited most of the participants. SR, DM, and AW revised the manuscript. All authors listed have made a substantial, direct and intellectual contribution to the work, and approved it for publication.

### Conflict of Interest

The authors declare that the research was conducted in the absence of any commercial or financial relationships that could be construed as a potential conflict of interest.

## References

[1] WHO CANCER TODAY-International Agency for Research on Cancer (GLOBOCAN 2012). (2017). Available online at: http://gco.iarc.fr/today/home

[2] Chen W Zheng R Zhang S Zeng H Zuo T Xia C. Cancer incidence and mortality in China in 2013: an analysis based on urbanization level. Chin J Cancer Res. (2017) 29:1–10. 10.21147/j.issn.1000-9604.2017.01.01 28373748 PMC5348470

[3] Chen W Zheng R Baade PD Zhang S Zeng H Bray F. Cancer statistics in China, 2015. CA Cancer J Clin. (2016) 66:115–32. 10.3322/caac.21338 26808342

[4] Rice TW Blackstone EH Rusch VW. 7th edition of the AJCC cancer staging manual: esophagus and esophagogastric junction. Ann Surg Oncol. (2010) 17:1721–4. 10.1245/s10434-010-1024-1 20369299

[5] He J Clinical Practice Guidelines for the Diagnosis and Treatment of Esophageal Cancer. Beijing: Peking Union Medical University Press (2013).

[6] van Hagen P Hulshof MC van Lanschot JJ Steyerberg EW van Berge Henegouwen MI Wijnhoven BP Preoperative chemoradiotherapy for esophageal or junctional cancer. N Engl J Med. (2012) 366:2074–84. 10.1056/NEJMoa1112088 22646630

[7] Wang X Wang AR Fan JC Li J Bao Y Wang Y. [Results of a screening program on high incidence area of esophageal cancer in Yanting Sichuan from 2006 to 2011]. Zhonghua liu xing bing xue za zhi. (2012) 33:784–7. 10.3760/cma.j.issn.0254-6450.2012.08.006 22967328

[8] Zhang M Li X Zhang S Chen Q Wang F Zhang Y. Analysis of effect of screening of esophageal cancer in 12 cities and counties of Henan province. Zhonghua yu fang yi xue za zhi. (2015) 49:879–82. 26813719

[9] di Pietro M Canto MI Fitzgerald RC. Endoscopic management of early adenocarcinoma and squamous cell carcinoma of the esophagus: screening, diagnosis, and therapy. Gastroenterology. (2018) 154:421–36. 10.1053/j.gastro.2017.07.041 28778650 PMC6104810

[10] Fagundes RB de Barros SG Putten AC Mello ES Wagner M Bassi LA. Occult dysplasia is disclosed by Lugol chromoendoscopy in alcoholics at high risk for squamous cell carcinoma of the esophagus. Endoscopy. (1999) 31:281–5. 10.1055/s-1999-122 10376452

[11] Goda K Dobashi A Yoshimura N Kato M Aihara H Sumiyama K. Narrow-band imaging magnifying endoscopy versus lugol chromoendoscopy with pink-color sign assessment in the diagnosis of superficial esophageal squamous neoplasms: a randomised noninferiority trial. Gastroenterol Res Pract. (2015) 2015:639462. 10.1155/2015/639462 26229530 PMC4502310

[12] Committee AT Thosani N Abu Dayyeh BK Sharma P Aslanian HR Enestvedt BK ASGE technology committee systematic review and meta-analysis assessing the ASGE Preservation and Incorporation of Valuable Endoscopic Innovations thresholds for adopting real-time imaging-assisted endoscopic targeted biopsy during endoscopic surveillance of Barrett's esophagus. Gastrointest Endosc. (2016) 83:684–98.e7. 10.1016/j.gie.2016.01.007 26874597

[13] Sami SS Dunagan KT Johnson ML Schleck CD Shah ND Zinsmeister AR. A randomized comparative effectiveness trial of novel endoscopic techniques and approaches for Barrett's esophagus screening in the community. Am J Gastroenterol. (2015) 110:148–58. 10.1038/ajg.2014.362 25488897 PMC4387566

[14] Wang CH Lee YC Wang CP Chen CC Ko JY Han ML. Use of transnasal endoscopy for screening of esophageal squamous cell carcinoma in high-risk patients: yield rate, completion rate, and safety. Digestive Endosc. (2014) 26:24–31. 10.1111/den.12053 23551305

[15] Arantes V Albuquerque W Salles JM Freitas Dias CA Alberti LR Kahaleh M. Effectiveness of unsedated transnasal endoscopy with white-light, flexible spectral imaging color enhancement, and lugol staining for esophageal cancer screening in high-risk patients. J Clin Gastroenterol. (2013) 47:314–21. 10.1097/MCG.0b013e3182617fc1 23059405

[16] Ross-Innes CS Debiram-Beecham I O'Donovan M Walker E Varghese S Lao-Sirieix P. Evaluation of a minimally invasive cell sampling device coupled with assessment of trefoil factor 3 expression for diagnosing Barrett's esophagus: a multi-center case-control study. PLoS Med. (2015) 12:e1001780. 10.1371/journal.pmed.1001780 25634542 PMC4310596

[17] Mallick R Patnaik SK Wani S Bansal A. A Systematic review of esophageal microRNA markers for diagnosis and monitoring of barrett's esophagus. Digest Dis Sci. (2016) 61:1039–50. 10.1007/s10620-015-3959-3 26572780

[18] Wan JCM Massie C Garcia-Corbacho J Mouliere F Brenton JD Caldas C. Liquid biopsies come of age: towards implementation of circulating tumour DNA. Nat Rev Cancer. (2017) 17:223–38. 10.1038/nrc.2017.7 28233803

[19] Kumar S Huang J Abbassi-Ghadi N Mackenzie HA Veselkov KA Hoare JM. Mass spectrometric analysis of exhaled breath for the identification of volatile organic compound biomarkers in esophageal and gastric adenocarcinoma. Ann Surg. (2015) 262:981–90. 10.1097/SLA.0000000000001101 25575255

[20] Rustgi AK El-Serag HB. Esophageal carcinoma. N Engl J Med. (2014) 371:2499–509. 10.1056/NEJMra1314530 25539106

[21] Abnet CC Arnold M Wei WQ. Epidemiology of esophageal squamous cell carcinoma. Gastroenterology. (2018) 154:360–73. 10.1053/j.gastro.2017.08.023 28823862 PMC5836473

