# Supplementary material for: The Current Situation of Esophageal Cancer Staging and Perioperative Strategies Determination in Central and Southern China: A Cross Sectional Survey
Source: Front Oncol. 2019 Oct 22;9:1098. doi: 10.3389/fonc.2019.01098 (PMC6817579; doi:10.3389/fonc.2019.01098)
Supplement: Supplementary file 1 [file Data_Sheet_1.PDF]

Questionnaire for the awareness of esophageal cancer TNM staging system and therapy plan decision making, 13th edition

1 Background information

The hospital you are from is:

A. Teaching hospital B. Non-teaching hospital

The department you are from is:

A. Thoracic surgery B. Oncology (or radiation therapy) C. Digestive disease

Level of your position is:

A. Professor B. Senior attending C. Junior attending D. Resident E. Intern

2 Questions

1. What's the latest edition of TNM staging system for esophageal cancer by now?

A. 2nd B. 3rd C. 6th D. 7th

2. When was this latest edition released?

A. 2002 B. 2009 C. 2010 D. 2015

3. How do you define supraclavicular lymph node metastasis?

A. Regional lymph node metastasis

B. Remote lymph node metastasis

C. depending on the location of primary tumor

4. How do you define celiac lymph node metastasis?

A. Regional lymph node metastasis

B. Remote lymph node metastasis

C. depending on the location of primary tumor

5. Which of the factors is not involved in the staging system for esophageal cancer?

A. Tumor invasion

B. Number of metastatic regional lymph node

C. Remote metastasis

D. Histologic cell type

E. Tumor length

F. Histologic grade

G. Location of tumor

6. The tumors that originate from the lower thoracic esophagus or invade the esophagogastric junction should be staged according to the esophageal cancer TNM staging system:

A. True B. False

7. The tumors that originate within the proximal 5 cm of the stomach (cardia) but didn't extend into the esophagogastric junction should be staged according to the esophageal cancer TNM staging system:

A. True B. False

8. How do you get access to the updates of TNM staging system for esophageal cancer?

A. Internet search engine

B. Literature

C. Wechat, Weibo, Facebook, Twitter and other self-media

D. Academic conference

E. Smart phone APP

F. Textbook

G. Other ways

9. Do you think the present edition is controversial?

A. Yes B. No

10. How many cycles of neo-adjuvant chemotherapy do you think is suitable for patients with esophageal cancer?

A. 2 B. 3 C. 4 D. 5

11. How many cycles of adjuvant chemotherapy do you think is suitable for patients with esophageal cancer after radical resection?

A. 2 B. 4 C. 6

12. Which do you think is the best chemotherapy plan for patients with esophageal squamous cell carcinoma after radical resection?

A. 5-Fu + platinum B. paclitaxel + platinum C. Others

13. What's the usually recommended dose of neo-adjuvant radiotherapy for patients with esophageal cancer?

A. 20Gy B. 40Gy C. 60Gy

14. What's the best therapy for esophageal squamous cell carcinoma patients with lymph node metastasis?

A. Neo-adjuvant chemoradiotherapy

B. Neo-adjuvant chemotherapy

C. Neo-adjuvant radiotherapy
